# Improving implementation of health promotion interventions for maternal and newborn health

**DOI:** 10.1186/s12884-017-1450-1

**Published:** 2017-08-31

**Authors:** H. J. Smith, A. G. Portela, C. Marston

**Affiliations:** 1 Centre for Maternal and Newborn Health, Liverpool School of Tropical Medicine, Liverpool, UK; 2 Department of Maternal, Newborn, Child and Adolescent Health, World Health Organization, Geneva, Switzerland; 3 Faculty of Public Health and Policy, London School of Tropical Medicine and Hygiene, London, UK

## Background

Recently published guidance from the World Health Organization (WHO) on health promotion interventions for maternal and newborn health (MNH) recommends a series of interrelated interventions to improve access to and use of skilled care during pregnancy, childbirth and after birth [1]. The recommendations are based on systematic reviews of available evidence on the effect of the interventions on maternal and newborn health outcomes, primarily care seeking outcomes. The recommendations can be grouped into different categories according to the strength of the recommendation as determined by an expert group and the applicability of the interventions to different contexts:

- Interventions which are strongly recommended that could be implemented in different contexts: including birth preparedness and complication readiness, male involvement, partnership with traditional birth attendants, culturally-appropriate skilled maternity care, companion of choice at birth, and community participation in quality improvement processes and in programme planning and evaluation interventions.
- Community mobilization through facilitated participatory learning and action cycles is strongly recommended and was studied through randomized controlled trials, which is a higher level of evidence than the other interventions. Its effect has been mainly studied in rural settings where access to health services is poor.
- One intervention, maternity waiting homes, was recommended to be implemented in contexts with limited access to services or for populations living in remote areas.
- Community-organised transport schemes were recommended only when it is not possible to organize more sustainable and reliable sources of transport.
- Two final interventions were considered research recommendations, because of the paucity of evidence found in the searches on care-seeking outcomes. However, the interventions are supported because of their potential to inform women about rights and because of their link to important human rights principles. Additional research and documentation is recommended to better understand how the promotion of awareness of human, sexual and reproductive rights/rights to access quality care and community participation in Maternal Death Surveillance and Response (MDSR) can affect care-seeking outcomes.

These health promotion interventions are largely designed to improve care seeking and home care practices across the care continuum (i.e. use of antenatal care, birth with a skilled attendant or facility birth, care with a skilled birth attendant or in a facility in case of complications or illness in women and newborns, and postnatal visits for woman and baby). The systematic reviews conducted to inform the recommendations prioritized collecting information on these outcomes as direct measures of intervention effectiveness.

Although the WHO guideline examines the effect of individual interventions, the experts who participated in the WHO guidance consultation emphasised that these outcomes are best addressed within a broad strategy that simultaneously tackles multiple factors affecting care-seeking and care practices in the home. Previous work from WHO has indicated that to optimize impact and build synergies, interventions should be conceived across different levels of the health system including individual, household, community, health services and policy [2]. For instance, an improvement in care-seeking for birth might require improving individual women’s knowledge about seeking skilled care; enhancing family and community support for skilled care; addressing structural factors such as transport to facilities; and simultaneously improving the quality of care women receive at facilities.

### Aim of the article collection

This series of papers, published together as a collection, ‘Factors that affect implementation of health promotion interventions for maternal and newborn health in low- and middle-income countries’, is aimed at those designing or implementing health promotion policies and programmes for maternal and newborn health. These papers provide details on the implementation strategies that complement existing WHO guidance. Details on implementation are reported in a structured way to help policymakers determine factors to take into account when considering whether and how an intervention can be implemented more effectively in their setting – information that is often missing from existing literature [3].

The article collection is based on background papers commissioned by WHO for the guideline development process. In addition to the systematic reviews of the effects of the interventions, WHO also commissioned papers to summarise the contextual factors that influenced implementation. The aim was to understand the context in which the interventions had been implemented and whether implementation and effectiveness might vary by context. Although not included in the guideline, WHO also commissioned a systematic review as well as a paper on the implementation of demand-side financing interventions [4], included in this series.

### Methods for the series

We used the ‘Supporting the Use of Research Evidence’ (SURE) framework to ensure consistency between papers and to provide a structured way to present the information, targeted to those charged with implementing interventions or designing policy. SURE is a multi-agency collaboration that supports policy makers and decision makers, aiming to ensure that decisions about health systems are well-informed by research evidence [5]. One of the SURE guides includes a checklist for identifying barriers to policy implementation. The papers in this series analyse each intervention according to the items in the SURE checklist (including stakeholders’ perceptions and experiences of the intervention, health service delivery and social and political contextual factors that may influence implementation), including additional items as necessary. Where possible, the papers also provide specific analysis of how implementation factors relate to care seeking for maternity care services – a priority outcome for these maternal and newborn health promotion interventions.

Each paper in the series examines one health promotion intervention from the WHO guideline. Authors conducted secondary analyses of studies that were identified in the corresponding systematic reviews and sometimes through supplementary searches. They extracted information on implementation factors using the SURE checklist categories. In some cases, studies retrieved in the systematic reviews that had not met the inclusion criteria for the review of effectiveness are nevertheless included here because they contained information about implementation. The authors included papers that mentioned or described any factors related to implementation of the intervention, in the results and/or in the discussion sections.

## Implementation considerations

Four groups of factors were identified that were common to the implementation of the interventions considered in this series. They are summarised below (individual and family level considerations, community support, factors relating to the provision of care at facilities and health system requirements). These factors, when appropriately considered within an intervention, can act as facilitators; or conversely when not given adequate attention in some settings may impede both implementation of interventions and achievement of the desired outcomes.

### Individual and family level

Tailoring implementation strategies to existing care practices and social norms is essential for programme success. For example, prevailing beliefs about pregnancy and birth being a normal process without risk affected use of maternity waiting homes [6]. These norms, along with the extent to which the causes of complications were recognised and understood, also affected birth and complications preparedness [7].

The influence of family and community decision makers is crucial in implementing interventions. For example, influential family or community decision makers affected women’s use of maternity waiting homes [6], engagement with birth preparedness and complication readiness interventions [7], how awareness of rights was promoted and supported [8] and uptake of demand-side financing interventions [4]. They also affected interventions to involve men in maternal and newborn health care [9]. Gender norms were also crucial in understanding implementation success. Interventions that took account of cultural norms around birth and integrated them [10], and interventions that women perceived as culturally appropriate and acceptable for birth, were integral to increasing acceptability and motivation to use childbirth facilities [6].

### Community support

It is important to engage not only with women of reproductive age and their families, but also with other members of their communities to develop an enabling environment, including fostering changes in broader social norms to improve care practices. Ways to encourage this engagement included using mass media to raise awareness or mobilize communities, and involving communities in developing materials such as leaflets used in health promotion interventions. These were reported to have helped create positive community perceptions of interventions [7, 8], and increased awareness and uptake of demand-side financing programmes [4]. Interventions reported to improve care seeking outcomes often included social mobilisation activities to generate community support, including partnerships with traditional birth attendants [11] and members of the broader community such as religious leaders or men [8].

Engaging the wider community can enable shared understandings and solutions around health problems to be developed and implemented. For instance, religious and community leader engagement was found to be crucial in developing new roles for traditional birth attendants [11] and in promoting the right to maternity care services [8]. Participation of family members and others in the community to improve birth preparedness and complication readiness was reported to have helped community members to make more preparations for childbirth, and to have contributed to an enabling environment that – according to the studies reviewed – increased awareness of maternal deaths, increased the sense of responsibility for maternal health in the community, and encouraged community members to interact directly with government officials to try to improve services [7, 8]. Involving the community helped identify factors limiting use of the maternity waiting homes, such as requiring approval or support of husbands and mothers in law to use waiting homes, or the specific ways in which women’s childcare or household duties prevented them leaving home for the extended periods implied by a stay in a maternity waiting home [6].

Attempts to address power imbalances and improve the voice of seldom heard groups are described in three papers in this series ([8–10]). Social inequalities within communities made it harder to reach marginalized women and to build common ground on which to develop awareness about women’s rights to maternity care services [8]. One intervention attempted to include those who are seldom heard by conducting separate focus groups for women and young people so they could raise issues salient to them [12]. The paper summarising approaches to raise awareness of rights [8] emphasises the importance of investing time in developing a common language, mutual aims and expectations and clarifying rules to address power imbalances.

Fostering local leadership instead of simply conducting consultative forms of participation can facilitate implementation of interventions. Attempts to cultivate deeper community participation in developing, implementing and monitoring culturally- appropriate care interventions may help give communities ownership and a stake in the success of those interventions [10], as well as building mutual respect [13]. Difficulties reported in fostering local leadership include unfair recruiting practices, particularly for paid roles. For instance, community workers employed to facilitate demand-side financing interventions were reported in one study to be family members appointed by local politicians and community leaders even though those family members were unlikely to want to perform the necessary duties [4].

### Provision of care at health facilities

Many of the interventions involve influencing healthcare worker attitude or behaviour, introducing new working relationships among different cadres of staff, or making organisational changes such as changes in facility policies or practices.

Addressing the attitudes of skilled healthcare workers towards interventions was important. This was reported, for instance, when establishing new working relationships between skilled and traditional birth attendants in order to tackle the negative attitudes some skilled attendants had towards their counterparts [11]. Similarly, there was initially lack of acceptance among nurses and midwives and concern about the role of a companion of choice at birth [14], and negative attitudes of healthcare workers in interventions to provide culturally-appropriate maternity care services [10].

Ensuring healthcare workers are clear about the intervention and their role in implementation is also key. Training healthcare workers on the benefits of the intervention as well as training to enhance their communication skills seemed to support implementation of both male involvement interventions [9] and interventions to encourage women to have a companion of choice at birth [14]. Formalising the roles and responsibilities for traditional birth attendants helped them be accepted by both skilled health workers and communities [11]. For one type of demand-side financing intervention – voucher schemes – healthcare workers in participating government and private facilities reported gaining skills and experience, making investments in facility infrastructure and being able to hire more staff as a result of implementing the intervention [4]. Efforts to elicit health providers’ buy-in included undertaking a situation analysis to understand their working context [8].

For several interventions, the quality of care provided at facilities posed a significant barrier to implementing interventions aiming to increase the number of women using those facilities. This was particularly the case for maternity waiting homes, where barriers to use were related to negative perceptions about the care that women and their families might receive in the facility [6]. Facility staff may also find it difficult to maintain quality in the face of increased demand for services [4] or respond in ways that affirm rights if there is increased enforcement of service delivery targets at the same time [8]. The act of implementing some of these health promotion interventions appeared to have a positive influence on healthcare workers and the organisation of care at facilities, which in turn supported implementation. It is challenging to encourage women to receive care where there is perceived poor quality, and unethical where quality is genuinely very poor [4].

There are examples where community participation in interventions contributed to efforts to increase provider accountability, for instance when individuals from communities are members of health facility committees [15] or where service statistics are publicised [16]. Providers who do not receive sufficient institutional support such as allowing time for participatory activities and rights-based elements of care, however, are unlikely to prioritise these types of interventions [8, 17]. Inaccessibility of facilities is an important barrier to implementation of health promotion interventions: for example, good accessibility of facilities seemed to improve community willingness to embrace interventions to improve birth preparedness and complication readiness [7]. Uptake of demand-side financing schemes was also affected by facility accessibility, especially if women faced long journeys to reach facilities [4]. Similarly, interventions to make services culturally appropriate can only be successful if they also address quality as well as broader access barriers [10].

### Health system requirements

The contextual evidence summarised in the series highlights how these complex and multifaceted interventions require adequate accompanying investment in health services and systems. For example, sustainable funds and policy changes are needed to integrate traditional birth attendants, including any remuneration needed to encourage them to take on new roles and integrate them into the formal health system [11]; some interventions such as maternity waiting homes require infrastructure upgrading or restructuring ([6, 9, 14]; and most interventions rely on concurrent government investment in the health system to ensure adequate services, quality care, and adequate levels of trained staff at facilities. Without these, women will remain reluctant to use services. In some contexts, for example where interventions coincide with civil war, political unrest, or where health systems are compromised, implementation will be even more difficult [9]. For example, in countries experiencing war or political unrest absence of or compromised basic infrastructure as well as security issues make it more unlikely that the required health system structure will be in place or investments made to improve it [9].

## Discussion

It is difficult to piece together the characteristics of successful implementation based on the effectiveness studies we retrieved from the comprehensive database searches in the original reviews. However, the barriers to implementation identified in this series reflect barriers to care seeking identified in a recent qualitative evidence synthesis on access to facility-based childbirth [18]. The same factors that interact to affect both access to and use of services also affect the implementation of interventions designed to improve access to and use of those services: it is vital to ensure that health promotion interventions are designed within a broad strategy that simultaneously tackles multiple factors and takes multiple levels of the health system into account.

One clear finding across the papers in this series is that implementation of interventions can be enhanced by using a participatory approach including dialogue to ensure that the perspectives of women, families, communities, and providers are incorporated. A participatory approach can help ensure interventions are designed appropriately and that implementation responds to feedback and the context. Although the included studies rarely provided information about sustainability, the material resources required for implementation, or the social relationships and histories of communities that might help or hinder implementation and underlying participatory approaches, there are several key characteristics policy makers should consider in implementing these interventions.

First, socio-political support and commitment is essential for success and sustainability of the health promotion interventions and for any participatory approaches that underlie them. Appropriate resources must be provided, including payments for training and ongoing support, as well as other resources, such as support from the wider community. Processes at the district or primary level also need to be supported by legal mandates and functional decentralization.

Second, health promotion approaches to increase use of maternity care services should be implemented in combination for synergistic effects. For instance, if community involvement helps lead to implementation of culturally-appropriate services, those services may be used by more people who in turn may have ideas for improvement which may help make services more efficient, or ensure people use services more effectively, thus reducing costs for the overall system. Conversely, if quality is poor, a programme cannot ethically promote the use services that might put women at risk and women are likely to spread the word about any poor treatment they receive.

Third, it is important to include representation of all key community groups and ensure the voices of women and members of minority groups are not lost, so that participatory interventions do not simply reinforce harmful power imbalances. Genuinely participatory approaches that make efforts to include disadvantaged groups can help create an enabling environment for those groups to be seen as legitimate participants, and be supported by others in what they do. To achieve meaningful participatory processes, it is essential to help communities and providers develop the skills they need to engage in dialogue, as well as to find ways to maintain dialogue once it has started [8, 19]. For this, wider socio-political support is essential.

Fourth, the effect of time is considered within two of the papers [8, 19]. It is important to allocate appropriate time to engage participants and develop collaborative relationships to improve health [19]. It is also important to take account of the broader time dimensions of interventions. Over time, for instance, participants in collaborative processes can develop trust in each other and a sense of their own roles, as well as ‘learning together’ how to engage in productive dialogue [8, 19]. Time is also important during political transitions such as when government officials change, with new officials needing to be updated on the project and new relationships needing to be developed [8, 19].

A health promotion intervention and a participatory and dialogue-based approach are not in and of themselves ‘empowering’ or ‘best practice’. It is noticeable that very few of the studies reported or addressed any negative or potentially negative aspects of the interventions they describe, or their implementation.

### Limitations in the evidence presented in the series

The series is based on studies identified through systematic reviews designed primarily to investigate the effectiveness of interventions, and not specifically to illuminate barriers and facilitators to implementation. Details were often limited and key implementation considerations not always described. In general, the studies did not provide detailed descriptions of the interventions or of implementation. Few of the studies explicitly collected data on stakeholder preferences or attitudes towards the interventions. Certain implementation themes were rarely addressed including resource implications and intervention integrity; other contextual issues such as political leadership, donor policies or existing legislation were also rarely described. Included studies rarely provided information about sustainability, the material resources required for implementation, or the social relationships and histories of communities that might help or hinder participatory approaches. Large parts of the data analysed in this series were drawn from the background and discussion sections of the included studies, often based on the study authors’ reflections rather than on specific empirical examples. Finally, the papers in this series share the limitations of most reviews, in that some studies – unpublished studies in particular – may have been missed.

## Conclusion

In this series we examine individual health promotion interventions, yet, as mentioned already, programme designers must consider multiple strategies to simultaneously address the factors that affect care-seeking and care practices in the home. Other authors have grappled with how best to ensure implementation of complex interventions is ‘as right as feasibly possible’ in real-world conditions [20], and some suggest that for interventions that are unsuccessful, research-based evidence should help determine whether “the intervention was inherently faulty (that is, failure of intervention concept or theory), or just badly delivered (failure of implementation)” [21]. We were unable to make these assessments based on the papers in this series. Nevertheless, the papers propose that interventions may often not achieve their desired objectives because of lack of attention to important issues at the design and implementation stages and because of failure to engage with intended beneficiaries and the wider community. With robust formative research, including careful consideration of the contextual factors highlighted in this series, programmes will be better able to implement interventions that improve access to and use of skilled care, during pregnancy, childbirth, and after birth. A participatory approach that encourages dialogue between different actors including women, men, community members and health providers appears to be an important feature for success.

To assess the evidence on the effectiveness of interventions, expert groups require a better understanding of programme implementation and context. A standardized way of reporting on context and processes throughout the programme would allow for easier synthesis of this information, and facilitate communication between research and practice. Such reporting should also contain information on the changing relationships between different actors, processes and structural factors [22]. A tool for better reporting of implementation issues has been developed, including ways to report dynamic contextual factors in programmes and research [23]. This type of information in addition to information on health outcomes will help deepen our understanding of how programmes develop over time and address challenges such as how variations in contexts and conditions can affect programme success, helping improve programmes in the future.
