# Correction to: Improving implementation of health promotion interventions for maternal and newborn health

**DOI:** 10.1186/s12884-018-1719-z

**Published:** 2018-04-30

**Authors:** Helen Smith, Anayda Portela, Cicely Marston

**Affiliations:** 1 Centre for Maternal and Newborn Health, Liverpool School of Tropical Medicine, Liverpool, UK; 2 Department of Maternal, Newborn, Child and Adolescent Health, World Health Organization, Geneva, Switzerland; 3 Faculty of Public Health and Policy, London School of Tropical Medicine and Hygiene, London, UK

## Correction

Following publication of the original article [1], it was noted that the formatting of the authors’ manes was inconsistent with that of the other articles in the series. The names are currently set out as initial.last name whilst the other papers give the authors’ full names. The names of the authors should therefore read as follows:

Helen Smith, Anayda Portela and Cicely Marston

The full, correct author list can also be seen in this correction.
